# Distribution, source, water quality and health risk assessment of dissolved heavy metals in major rivers in Wuhan, China

**DOI:** 10.7717/peerj.11853

**Published:** 2021-07-27

**Authors:** Xingyong Zhang, Qixin Wu, Shilin Gao, Zhuhong Wang, Shouyang He

**Affiliations:** 1 Key Laboratory of Karst Geological Resources and Environment, Ministry of Education, Guizhou University, Guiyang, Guizhou, China; 2 The College of Resources and Environmental Engineering, Guizhou University, Guiyang, Guizhou, China; 3 School of Public Health, Key Laboratory of Environmental Pollution and Disease Monitoring of Ministry of Education, Guizhou Medical University, Guiyang, Guizhou, China

**Keywords:** Heavy metals, Source, Heavy metal pollution index, Health risk assessment, Wuhan

## Abstract

Heavy metals are released into the water system through various natural processes and anthropogenic activities, thus indirectly or directly endangering human health. The distribution, source, water quality and health risk assessment of dissolved heavy metals (V, Mn, Fe, Co, Ni, Zn, As, Mo, Sb) in major rivers in Wuhan were analyzed by correlation analysis (CA), principal component analysis (PCA), heavy metal pollution index (HPI), hazard index (HI) and carcinogenic risk (CR). The results showed that the spatial variability of heavy metal contents was pronounced. PCA and CA results indicated that natural sources controlled Mn, Fe, Co, Ni and Mo, and industrial emissions were the dominant factor for V, Zn and Sb, while As was mainly from the mixed input of urban and agricultural activities. According to the heavy metal pollution index (HPI, ranging from 23.74 to 184.0) analysis, it should be noted that As and Sb contribute most of the HPI values. The health risk assessment using HI and CR showed that V and Sb might have a potential non-carcinogenic risk and As might have a potential carcinogenic risk to adults and children in the study area (CR value exceeded target risk 10^−4^). At the same time, it was worth noting that As might have a potential non-carcinogenic risk for children around QLR (HI value exceeded the threshold value 1). The secular variation of As and Sb should be monitor in high-risk areas. The results of this study can provide important data for improving water resources management efficiency and heavy metal pollution prevention in Wuhan.

## Introduction

With the rapid development of the social economy, industry, agriculture, medicine, and mining activities are increasing, which have seriously affected the quality of surface water worldwide (Gao et al., 2020; Wang et al., 2017; Zeng, Han & Yang, 2020). Heavy metals have the characteristics of toxicity, persistence and bioaccumulation, which cause environmental pollution to the water ecosystem and threaten human health (Cameron, Mata & Riquelme, 2018; Zaric et al., 2018; Zeng et al., 2019). There are two main exposure pathways (direct ingestion and dermal absorption) of heavy metals in water bodies (Gao et al., 2020; Giri & Singh, 2014). However, aquatic foods and plants as human food sources may also lead to the exposure of heavy metals in water (Krishnamurti et al., 2015; Qu et al., 2018). Therefore, due to the bioaccumulation of heavy metals, even if humans do not directly drink or contact water contaminated by heavy metals, they will also be exposed to high levels of heavy metals from food (Antoniadis et al., 2016; Jiang et al., 2016). Thus, the pollution of heavy metals in the water environment has been widely concerned worldwide (Ali et al., 2016; Chowdhury et al., 2016; Zeng & Han, 2020a; Zeng & Han, 2020b).

Heavy metals enter the water body mainly through natural processes (atmospheric dry/wet deposition, rock weathering and volcanism) and anthropogenic activities (industrial, agricultural, medical and urban sewage) (Han & Liu, 2004; Han et al., 2019; Krishna, Satyanarayanan & Govil, 2009; Li & Zhang, 2010; Meng et al., 2016). The metal availability of organisms is determined by the concentration and species of heavy metals in water. Therefore, more attention should be paid to heavy metals in surface water because these heavy metals may affect human health through food, water and body contact (Qu et al., 2018). The study of the concentration, distribution, source, water quality and health risk levels of dissolved heavy metals in surface water is the basis for the effective control of water pollution and the maintenance of water ecosystem health (Xiao, Jin & Wang, 2014; Xiao et al., 2019).

Correlation analysis (CA) and principal component analysis (PCA) are common tools for studying heavy metals in water environment. They can better identify the natural or anthropogenic sources of heavy metals (Gao et al., 2020; Giri & Singh, 2014; Qu et al., 2018; Thuong et al., 2013; Wang et al., 2017; Xiao et al., 2019). Furthermore, heavy metal pollution index (HPI) was used to analyze the combined effects on water quality (Tiwari et al., 2015). Hazard quotient (HQ), hazard index (HI) and carcinogenic risk (CR) are commonly used to assess human health risks of heavy metals (Qu et al., 2018; Wang et al., 2017; Wu et al., 2009b; Zeng et al., 2019). Moreover, geographical information system (GIS) is also used to show the spatial distribution of pollutants (Qu et al., 2018; Tiwari et al., 2015).

With the growth of population and the rapid development of agriculture, cities and industries in the past decades, rivers in Wuhan have been polluted by toxic heavy metals (Gong et al., 2018; Xu et al., 2020a). Therefore, the sources and health risks associated with heavy metals in these rivers need to be explored to protect water resources and improve water quality. Heavy metal pollution in surface water and sediment in Wuhan has been studied a lot (Du et al., 2014; Hu et al., 2012; Yang et al., 2016). However, the systematic study on geochemical characteristics, sources, water quality and health risks of dissolved heavy metals in surface rivers of Wuhan is still lacking, especially in the analysis of spatial distribution and potential health hazards of heavy metals in surface rivers through different exposure pathways and source identification.

In this study, water from surface rivers in Wuhan was collected systematically. Nine dissolved heavy metals in the river water were analyzed, with the following objectives: (1) to understand the spatial distribution of heavy metals in surface rivers; (2) to explore the potential sources of heavy metals; (3) to evaluate river water quality and the risk level of heavy metals to human health, and to map their spatial distribution. The results can provide important information for water resources management and prevention and control of heavy metal pollution to reduce the health hazards to the public.

## Materials and Methods

### Study area

**Figure 1:**
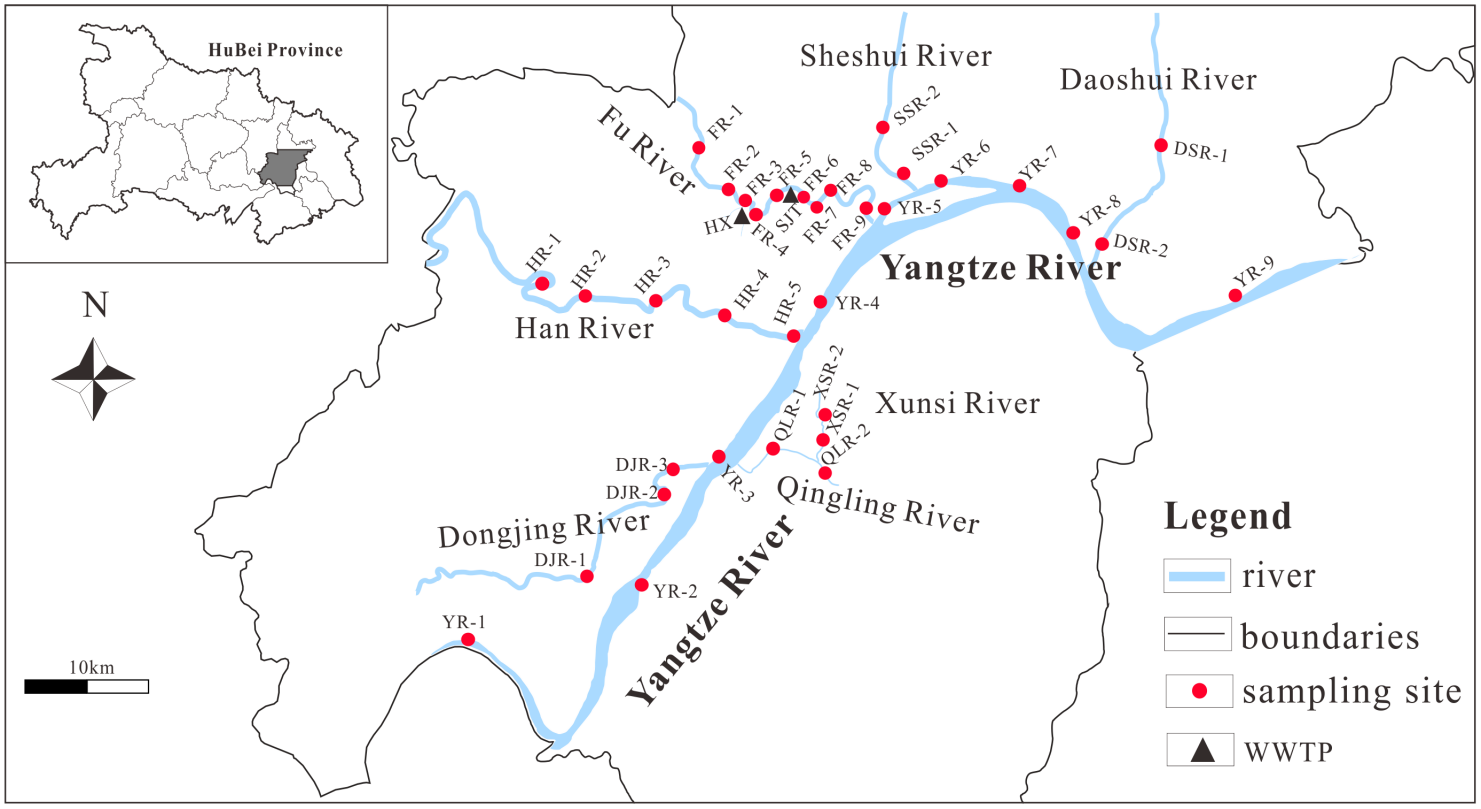
Sample sites distribution of the studied rivers in Wuhan.

Wuhan is located in the central part of China and the middle reaches of the Yangtze River basin (29°58′-31°22′N; 113°41′-115°05′E), which is the capital of Hubei Province and the central city of Yangtze River Economic Zone. The landform belongs to the transition region from the southeast hills of Hubei through the eastern margin of Jianghan Plain to the lower elevations at the southern foot of the Dabie Mountains. The climate of Wuhan belongs to the north subtropical monsoon climate, with a mean annual temperature of between 15.8 °C and 18.5 °C. The annual precipitation ranges from 1,150 to 1,450 mm, of which about 40% occurs in the rainy season (June to July). With a developed water system and rivers, Wuhan has formed a complex and unique water area with a total water area of 2,217.6 km^2^ (Xu & Wang, 2020). Eight representative rivers are selected in this study, which are Yangtze River (YR), Dongjing River (DJR), Han River (HR), Qingling River (QLR), Xunsi River (XSR), Fu River (FR), Sheshui River (SSR) and Daoshui River (DSR) (Fig. 1). The lithology of the basin is mainly carbonate rock (Guan, Meng & Zhou, 2008). Wuhan industry is developed, including automobile, chemical, steel, metallurgy, *etc*. Heavy metal pollution in rivers is becoming more and more severe due to the long-term acceptance of untreated domestic and industrial wastewater (Li, 2019).

### Sample collection and analysis

In July 2019, 34 surface water samples were collected from eight rivers in Wuhan, of which YR1~9 HR1~5, DJR1~3, FR1~9, XSR1~2, QLR1~2, DSR1~2, SSR1~2 (Fig. 1), which reasonably reflected the water quality of surface rivers in Wuhan. After collection, the samples were immediately filtered with 0.22 μm acetate fiber filter membrane and acidified with ultra-pure concentrated HNO_3_ to pH < 2, then sealed in a pre-pickled polyethylene bottle and stored in a 4 °C freezer for the determination of heavy metals. The remaining unfiltered water samples were acidified with sulfuric acid to pH < 2 to determine total phosphorus and total nitrogen. The pH, electrical conductivity (EC) and dissolved oxygen (DO) were measured by portable water quality analyzer (WTW Company, Weilheim, Upper Bavaria, Germany). Nine heavy metal elements (V, Mn, Fe, Co, Ni, Zn, As, Mo and Sb) in water samples were determined by inductively coupled plasma-mass spectrometry (ICP-MS, NexION300X) of the Institute of Geochemistry, Chinese Academy of Sciences. Standard reference materials (GSB04-1767-2004) were used for method validation and quality control (Liu & Han, 2021; Xu et al., 2020a; Zhang et al., 2018). All samples, standards and program blanks of the same batch of samples were analyzed. The recoveries ranged from 90.0% to 110.4%. The relative standard deviation of heavy metals is ± 5%.

### Statistical analysis

In this study, correlation analysis (CA) and principal component analysis (PCA) were used to identify pollution sources. PCA is a common statistical analysis method in studying the sources of heavy metal pollution (Loska & Wiechuła, 2003; Zeng, Han & Yang, 2020). In the PCA analysis of the data, the feasibility of the data was tested by Kaiser–Meyer–Olkin (KMO) and Bartlett’s sphericity test (*p* < 0.001) (Varol, 2011). The principal components were extracted only when the eigenvalue is greater than or equal to 1 (Kowalkowski et al., 2006). All data were processed by SPSS 22.0.

### Heavy metal pollution index

Heavy metal pollution index (HPI) can provide the comprehensive impact of a single heavy metal on the overall water quality. The HPI model (Mohan, Nithila & Reddy, 2008; Qu et al., 2018) is given by Eq. (1)

(1) }{}{\rm HPI}=\displaystyle{{\mathop \sum \nolimits_{{\rm i = 1}}^{\rm n} {\rm (}{{\rm Q}_{\rm i}}{{\rm W}_{\rm i}}{\rm )}} \over {\mathop \sum \nolimits_{{\rm i = 1}}^{\rm n} {{\rm W}_{\rm i}}}}

(2) }{}{{\rm Q}_{\rm i}}=\displaystyle{{{{\rm c}_{\rm i}}} \over {{{\rm s}_{\rm i}}}}{\rm \times 100}

(3) }{}{{\rm W}_{\rm i}}=\displaystyle{{\rm k} \over {{{\rm s}_{\rm i}}}}

where W_i_ is the unit weight of the ith parameter, which can reflect its importance. Q_i_ is the sub-index of the ith heavy metal parameter, and n is the number of parameters selected in the study. c_i_ is the concentration of the ith heavy metal parameter (μg/L), and s_i_ is the highest allowable value of the ith parameter in drinking water standard. The value of s_i_ in this study is from the World Health Organization (WHO) drinking water quality guidelines (WHO, 2011). Where k is the proportional constant, here we take K as “1”, which is convenient for calculation (Wanda, Gulula & Phiri, 2012). Now we typically use the modified scale to describe heavy metal pollution: low (HPI values < 15), medium (HPI values within 15–30) and high (HPI values > 30) (Tiwari et al., 2015).

Geographic information system (GIS) is widely used to collect various spatial data to express spatial variation (Gupta & Srivastava, 2010; Qu et al., 2018; Tiwari et al., 2015). Therefore, GIS technology was used to present the results of the HPI assessment of river heavy metal ecological risk in Wuhan. The HPI values of the sampling points were inserted into the surface rivers of Wuhan by inverse distance weighted (IDW) method to show the spatial distribution. A spatial distribution map was compiled by ArcGIS 10.7 software.

### Human health risk assessment model

Heavy metals are difficult to remove in water, and they can be recycled and bioaccumulated through a biochemical reaction process. Therefore, people need to pay attention to their harmful effects on human health and environmental impact on aquatic ecosystem (Waalkes, 2000; Wang et al., 2017; Wu et al., 2009a). Therefore, to facilitate the determination of appropriate management measures, the toxicity of heavy metals in the water system is worth studying (Lemly, 1996). In this study, hazard quotient (HQ) and hazard index (HI) were used to assess the health risk of heavy metals in rivers of Wuhan (Meng et al., 2016). Heavy metals in the water environment enter human body mainly through direct ingestion and dermal absorption (Giri & Singh, 2014; Zeng et al., 2019). HQ is estimated by the ratio of exposure to reference dose (RfD) in various pathways. HI is the sum of the HQs of the two main exposure pathways mentioned above, which can be used to represent the total potential non-carcinogenic risk of individual heavy metals. When HQ/HI is greater than 1, the pollution level may cause harm to human health, and there is a non-carcinogenic risk, which needs particular attention; however, when HQ/HI is less than 1, there is no health risk (Tripathee et al., 2016; Yi, Yang & Zhang, 2011). The calculation formula of HQ and HI is as follows:

(4) }{}{\rm AD}{{\rm D}_{{\rm ingestion}}}={\rm (}{{\rm C}_{\rm w}}\times{\rm  IR} \times{\rm  EF} \times{\rm  ED}{\rm )}{\rm /}{\rm (}{\rm BW \times AT}{\rm )}

(5) }{}{\rm AD}{{\rm D}_{{\rm dermal}}}={\rm (}{{\rm C}_{\rm w}} \times{\rm SA  }\times{{\rm K}_{\rm p}}\times{\rm  ET} \times {\rm EF} \times {\rm ED} \times 1}{{\rm 0}^{{\rm - 3}}}{\rm )}{\rm /}{\rm (}{\rm BW \times AT}{\rm )}{\rm \; }

(6) }{}{\rm HQ} ={\rm  ADD/RfD\; }

(7) }{}{\rm \; Rf}{{\rm D}_{{\rm dermal}}}={\rm  RfD \times AB}{{\rm S}_{{\rm GI\; \; \; \; \; }}}\;

(8) }{}{\rm HI  }=\sum {\rm HQs}

where ADD_ingestion_ is the average daily doses of direct intake (μg/kg/day); ADD_dermal_ is the average daily doses of skin absorption (μg/kg/day); C_w_ is the concentration of heavy metals in each water sample (μg/L); IR is the ingestion rate (L/ day); EF is the exposure frequency (days/year); ED is the exposure duration (years); AT is the average time (days); BW is the average body weight for adults and children (kg); ET is the daily exposure time (h/day); SA is the area of skin exposure (cm^2^); K_p_ is the dermal permeability coefficient in water (cm/h); RfD is the corresponding reference dose (μg/kg/day); ABS_GI_ is the gastrointestinal absorption factor (dimensionless). The values of these parameters come from (United States Environmental Protection Agency, 2009; Wang et al., 2017; Wu et al., 2009b).

The carcinogenic risk of heavy metals is evaluated by Eq. (9). Carcinogenic risk represents the increased probability of cancer due to chemical exposure (Chen & Liao, 2006; Obiri et al., 2006). CR values in US-EPA are divided into three levels: (1) CR value less than 10^−6^ indicates negligible level; (2) 10^−6^ < CR < 10^−4^ is acceptable level; (3) CR > 10^−4^ indicates high cancer risk to human (Qu et al., 2018).

(9) \left\{ {\matrix{{{\rm CR = ADD \times SF, \; \; \; \; \; \; \; \; \; \; \; \; \; \; \; \; \; \; \; \;  \; \;ADD \times SF < 0}{\rm .01}} \cr {{\rm CR = 1 - EXP}\left( {{\rm - ADD \times SF}} \right){\rm ,\; \; \; \;   ADD \times SF} \ge {\rm 0}{\rm .01}} \cr } } \right.

In this study, carcinogenic and non-carcinogenic risk assessments were performed for As, which was carcinogenic to humans. For the other eight heavy metals (V, Mn, Fe, Co, Ni, Zn, Mo, Sb), only non-carcinogenic risk assessments were considered. The HI and CR values of the sampling points were interpolated into the surface rivers of Wuhan by the IDW method to show the spatial distribution of non-carcinogenic risk and carcinogenic risk.

## Results

### Kolmogorov–Smirnov test of data

The normal distribution of the data in this study was tested by Kolmogorov–Smirnov (K–S) statistics (Table 1). Results indicated that only vanadium and dissolved oxygen were normal distribution. It could be seen from the test results that the average concentration of heavy metals might be seriously affected by the abnormal values of water samples. Therefore, the median concentrations were used for calculation. However, arithmetic means were used for comparison because the values in the drinking water standards are generally given as arithmetic means.

**Table 1:** Concentrations of dissolved heavy metals (μg/L), pH, EC (μs/cm) and DO (mg/L) in rivers in Wuhan.

	Min	Max	Mean	Median	SD	K-S test^a^
V	0.54	3.20	1.91	1.85	0.64	0.200
Mn	0.00	7.66	0.90	0.16	1.88	0.000
Fe	8.62	21.97	12.80	12.18	2.77	0.008
Co	0.03	0.19	0.06	0.05	0.04	0.000
Ni	0.46	3.77	1.16	0.86	0.72	0.000
Zn	0.39	7.45	2.10	1.52	1.81	0.000
As	2.51	11.15	3.72	3.13	1.74	0.000
Mo	1.07	6.00	2.28	1.73	1.26	0.000
Sb	1.14	16.29	4.29	4.07	2.73	0.100
pH	5.51	9.14	7.96	7.95	0.75	0.011
EC	213.00	661.00	344.00	329.03	92.71	0.013
DO	2.59	19.24	7.75	6.82	3.26	0.540

**Note:**

a Kolmogorov–Smirnov test.

### Physicochemical characteristics

The water quality parameters (pH, EC, DO) of water samples are exhibited in Table 1. The pH value of the river water ranged from 5.51 to 9.14, with a mean of 7.96, demonstrating slight alkalinity characteristics. The DO ranged from 2.59 to 19.24 mg/L, with a mean of 7.75 mg/L. The EC ranged from 213.0 to 661.0 μS/cm, with a mean of 344.0 μS/cm. In a word, pH, EC and DO had pronounced variability in rivers in Wuhan.

### Concentrations of heavy metals in river water

The concentrations of dissolved heavy metals in rivers in Wuhan are demonstrated in Table 1. V, Fe, Ni, Zn, As, Mo and Sb had relatively high concentration, with mean concentrations of 1.91 μg/L, 12.80 μg/L, 1.16 μg/L, 2.10 μg/L, 3.72 μg/L, 2.28 μg/L, 4.29 μg/L, respectively. Moreover, there were a few elements whose concentration was less than 1 μg/L, represented by Mn (0.90 μg/L) and Co (0.06 μg/L). Fe, Sb and As were the most abundant metals in sampling periods.

Concentrations of dissolved heavy metals were compared with the limits for drinking water and surface water established by China EPA (2002, 2006), WHO (2011) and Environmental Protection Administration of China (2003) (Table 2). The total concentration (average) of all heavy metals in river water was within the three drinking water standard limits (except V, there is no corresponding standard). The concentrations of Zn and As belonged to Grade I of surface water standard, which indicated that the overall water quality of the Wuhan river water was excellent. However, Sb concentrations in YR and HR exceeded the limit values of the three drinking water standards and in FR was close to the limit. In addition, the As concentrations in DJR and QLR were significantly higher than those in other rivers, which were close to the standard limits of drinking water. These heavy metals exceeded or closed to guideline values could be considered as pollutants, which might be caused by relatively high anthropogenic input at the corresponding sampling points. For example, Sb concentration in HR and As concentration in QLR were higher than the drinking water guideline, which indicated that the two rivers were significantly affected by anthropogenic input.

**Table 2:** Comparison of heavy metal concentrations (μg/L) in rivers in Wuhan with water quality standards for drinking water and surface water.

		V	Mn	Fe	Co	Ni	Zn	As	Mo	Sb
Total		1.91	0.90	12.80	0.06	1.16	2.10	3.72	2.28	4.29
Yangtze River		1.83	0.29	11.66	0.03	0.81	1.93	3.05	1.44	5.15
Dongjin River		1.21	0.21	13.67	0.06	0.72	0.97	6.54	1.75	1.55
Qingling River		1.98	1.27	11.33	0.07	1.76	0.69	7.80	4.54	2.40
Xunsi River		1.88	3.59	17.97	0.17	2.13	1.68	4.05	4.58	2.62
Han River		2.93	1.78	14.70	0.04	0.89	4.32	3.02	3.62	6.84
Fu River		1.95	0.96	13.06	0.07	1.52	2.00	3.27	2.00	4.51
Sheshui River		1.28	0.04	10.51	0.06	0.83	1.33	2.89	1.26	3.41
Daoshui River		1.07	0.09	9.35	0.06	1.22	2.14	2.80	1.28	1.58
World average^a^		0.71	34.00	66.00	0.15	0.80	0.60	0.62	0.42	0.07
China^b^			100	300	1000	20	1000	10	70	5
WHO^c^			500	300		20	3000	10	70	5
US EPA^d^			500	300			5000	10		6
Grade^e^	I						50	50		
	II						1000	50		
	III						1000	50		
	IV						2000	100		
	V						2000	100		

**Notes:**

a Gaillardet, Viers & Dupré (2014).

b Chinese drinking water standards (GB 5749-2006).

c WHO (2011) drinking water guidelines.

d Environmental Protection Administration of China (2003) drinking water standards.

e Chinese surface water standards (GB 3838-2002).

Table S1 shows the comparison of some heavy metals in rivers in Wuhan with other rivers in the world. Except for Mn, Fe, and Co, the average concentrations of heavy metals were higher than the world average. The average heavy metals in rivers in Wuhan were lower than other rivers in China, such as Xiangjiang (Zeng et al., 2015), Dan River (Meng et al., 2016), Tarim River (Xiao, Jin & Wang, 2014), Zhujiang River (Zeng et al., 2019), and Huai River (Wang et al., 2017). The heavy metals concentrations in some developing countries were generally higher than in this study, such as Vietnam, Iran, Nigeria, India and Turkey (Eneji, Sha’Ato & Annune, 2012; Giri & Singh, 2014; Nasrabadi, 2015; Thuong et al., 2013; Varol & Şen, 2012). However, in this study, the average heavy metals were higher than in some developed countries, such as the USA, France, Australia, Canada (Elbaz-Poulichet et al., 2006; Gaillardet, Viers & Dupré, 2014; Markich & Brown, 1998; Warnken & Santschi, 2009). This showed that the pollution level of heavy metals in rivers in Wuhan was at a medium level.

### Spatial distribution of heavy metals

#### Spatial distribution of heavy metals in the main stream

**Figure 2:**
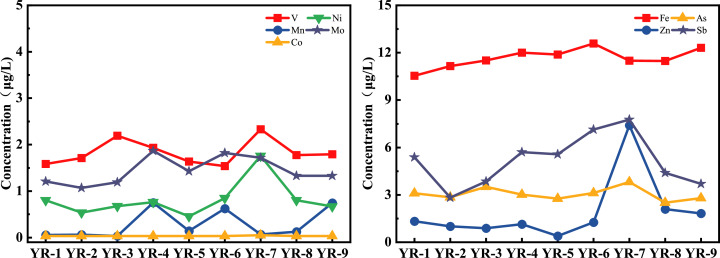
Spatial variations of nine heavy metals in nine sampling sites along the mainstream from Yangtze River in Wuhan.

The spatial distribution of heavy metals in the main stream is presented in Fig. 2. In general, Fe, Mn and Co concentrations presented a slowly increasing trend from upstream to downstream. In contrast, V, Ni, Mo, Zn, As, Sb increased slowly from upstream to downstream and then decreased gradually at YR-7. It should be noted that V, Ni, Zn, As and Sb concentrations were higher at YR-7, and Mn, Fe and Mo concentrations were higher at YR-4 and YR-6, while the fluctuation of Co along the main stream was not apparent. Different distribution of nine heavy metals might be affected by various local human activities, like industrial, agricultural, domestic, *etc*.

#### Spatial distribution of heavy metals in tributaries

**Figure 3:**
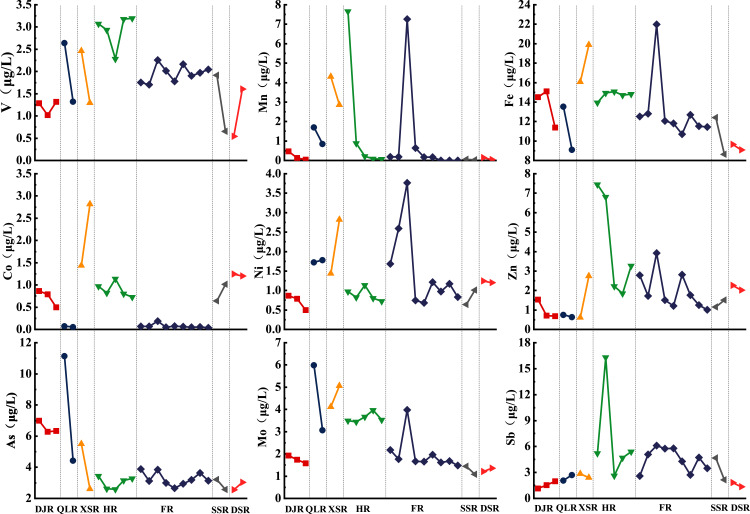
Spatial distribution of heavy metals in tributaries from Yangtze River in Wuhan.

The spatial distribution of each heavy metal in tributaries is exhibited in Fig. 3. The concentrations of heavy metals in QLR, XSR, HR and FR were relatively high, while those in DJR, SSR and DSR were relatively low. Although the spatial distribution showed wide variation, according to the spatial distribution characteristics of heavy metals, we could conclude three main distribution patterns: (1) the V, Zn and Sb concentrations in HR were higher than those in other rivers; (2) the Co, Fe, Mn, Ni and Mo concentrations in XSR were higher; (3) The concentration of As in QLR and DJR was higher. In addition, it was noteworthy that all heavy metals at FR-3 showed a solid upward trend, which might be related to the effluent of the Hanxi wastewater treatment plants upstream of the sampling point.

### Statistical analysis

**Figure 4:**
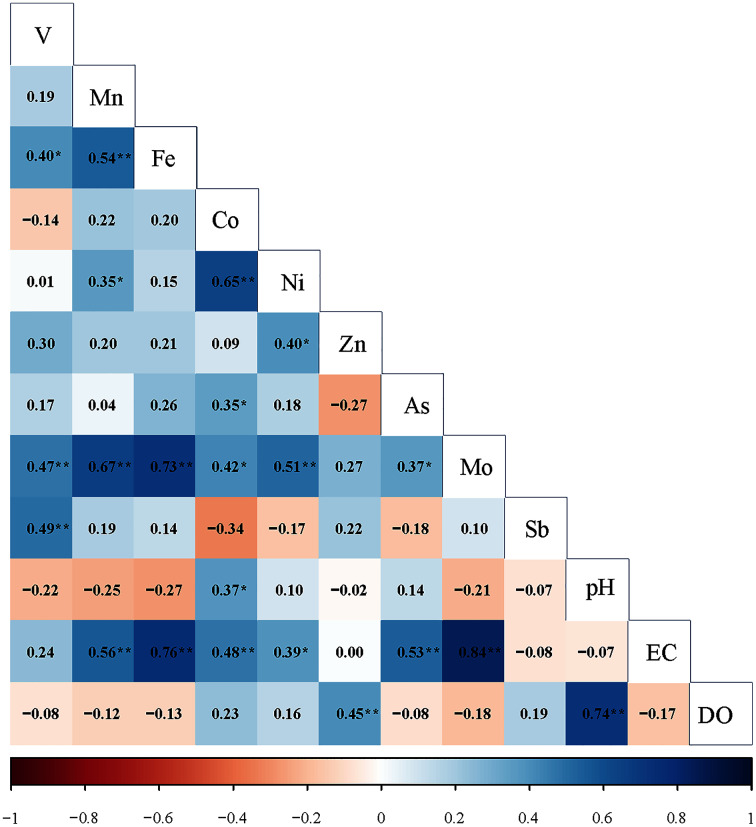
Correlation coefficients of heavy metals and physicochemical parameters (pH, EC and DO) in rivers in Wuhan; an asterisk (*) denotes *p* < 0.05; two asterisks (**) denotes *p* < 0.01.

Correlation matrix can show the correlation between survey variables by presenting the overall consistency of the data set (Chen et al., 2007; Helena, 2000; Wang et al., 2017). In this study, to identify the likely sources of heavy metals in the rivers of Wuhan, Spearman’s correlation analysis method was used to initially determine the degree of correlation between nine dissolved heavy metals. The results are shown in Fig. 4. Mn, Fe, Co, Ni and Mo were positively correlated, and the correlation coefficients ranged from 0.15 to 0.73. A significant positive correlation was observed between V and Sb (*p* < 0.01). In addition, Mo was also significantly positively correlated with V (0.47) and As (0.37).

**Figure 5:**
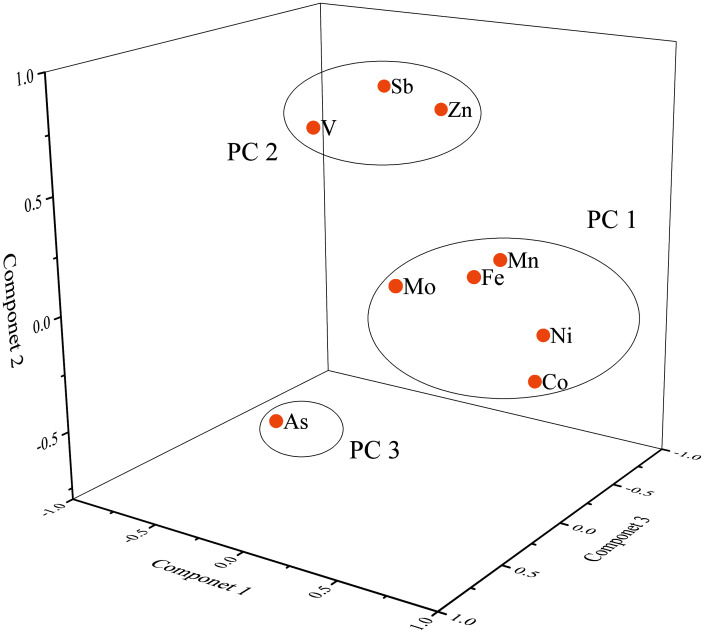
Loading plot of factors or dissolved heavy metals in rivers.

The application of PCA can more accurately identify the sources of these heavy metals (Dong et al., 2015; Kumar et al., 2017). According to the Kaiser–Meyer–Olkin (KMO) value (0.73) and Bartlett’s sphericity test (zero) results, the data in this study could be analyzed by PCA (Varol, 2011). Three principal components (PC) with eigenvalues > 1 (eigenvalue of 3.74 for PC1; 2.11 for PC2; and 1.19 for PC3) were extracted, explaining approximately 78.27% of the total variance. Three PCs were presented in Table 3 and Fig. 5. In PC1, the load of Mn (0.76), Fe (0.75), Co (0.92), Ni (0.88) and Mo (0.58) was higher, which explained the variance of 35.25%. The PC2 explained 25.81% of the total variance, mainly including V (0.80), Zn (0.78) and Sb (0.84). Finally, As (0.84) and Mo (0.66) had a high load in PC3, accounting for 17.21% of the total variance.

**Table 3:** Varimax rotated component matrix for dissolved heavy metals (the significance of KMO and Bartlett’s sphericity test is <0.001).

Eigenvalues	3.74	2.11	1.19
Variance (%)	35.25	25.81	17.21
Cummulative (%)	35.25	61.07	78.27
**Variable**	**PC1**	**PC2**	**PC3**
V	0.02	0.80	0.45
Mn	0.76	0.30	0.14
Fe	0.75	0.28	0.33
Co	0.92	−0.18	0.09
Ni	0.88	−0.03	−0.03
Zn	0.27	0.78	−0.17
As	0.08	−0.29	0.84
Mo	0.58	0.29	0.66
Sb	−0.06	0.84	−0.20

**Notes:**

Extraction method: Principal component analysis.

Rotation method: Varimax with Kaiser normalization.

## Discussion

### Controlling factors for dissolved heavy metals

The content of dissolved heavy metals in water is controlled by many factors, such as pH, EC and lithology (Gao et al., 2020; Xu et al., 2020b). The competition of relative binding sites between metal and hydrogen ions is affected by pH (Papafilippaki, Kotti & Stavroulakis, 2008). The lower the pH value, the more intense the competition. Moreover, the lower pH may dissolve metal complexes in sediments, thereby releasing free metal ions (Gao et al., 2020). However, in this study, pH was only significantly positively correlated with Co (0.365) (*p* < 0.05), but was no correlation with V, Mn, Fe, Ni, Zn, As, Mo and Sb (Fig. 4). Therefore, PH is not the primary control factor of dissolved heavy metal content in the study area. EC can measure the total dissolved metal ions in water and reflect the biogeochemistry and land use (Walker & Pan, 2006). Higher EC may lead to increased dissolved heavy metals in water (Gao et al., 2020). As shown in Fig. 4, EC was significantly positively correlated with Mn (0.557), Fe (0.757), Co (0.485), Ni (0.381), As (0.531) and Mo (0.845). The results showed that EC played an essential role in the content of dissolved heavy metals in the river.

Rainfall is an important factor in controlling dissolved heavy metals in rivers (Li & Zhang, 2010). Although it has a dilution effect in the rainy season, the amount of non-point source pollutants, mine wastewater and geological materials generated by weathering entering rivers may increase (Li & Zhang, 2010; Xu et al., 2020b). Temperature, rainfall and other factors will affect the chemical weathering of rocks (Chen et al., 2020; Ma et al., 2020; Xu et al., 2020b; Zhong et al., 2017). In the rainy season, with the increase of river flow, chemostatic behavior is induced by increasing the surface area of active minerals, which accelerates the rocks’ weathering (Xu et al., 2020b; Zhong et al., 2017). Therefore, the release rate of dissolved heavy metals in carbonate rocks will increase with the acceleration of rock weathering. In addition, under high flow conditions, the solute concentrations produced by weathering of different rocks may be changed by dilution due to the decrease of water-rock interaction time (Xu et al., 2020b; Zhong et al., 2017). In our study, the low concentrations of Fe, Mn, Co, Ni and Mo could be attributed to the dilution effect at high flow, which was speculated to be derived from natural processes. On the other hand, the relatively high concentrations of V, Zn, Sb and As could be considered the increase of anthropogenic emissions under rainfall.

### Potential source identification

The sources of heavy metals are diverse, and each element may have multiple sources. CA and PCA were used to identify the sources of nine elements in our study.

CA mainly represented the correlation between two elements, and PCA can divide elements into different arrays, which indicated different anthropogenic or natural sources of these elements (Gao et al., 2020; Zeng et al., 2015). The higher factor explained the total variance, the higher contribution of the source to the nine elements (Li, 2007). According to the CA and PCA results, three categories could be identified for the dominant sources of nine heavy metals in Wuhan.

Group 1 defined that Mn, Fe, Co, Ni and Mo were related (Fig. 4), which indicated that their sources and behavior might originate similarly. Mn, Fe, Co, Ni and Mo presented a robust positive loading in PC1 (Fig. 5). Fe was the central element in the earth’s crust and Mn, Co, Ni and Mo mainly originated from rock weathering and soil-forming processes (Li & Zhang, 2010; Xiao et al., 2019; Yokoo et al., 2004). Combined with significantly lower Mn, Fe and Co concentrations than the world average, we attributed group 1 to the geological source of the basin.

As shown in Table 3, V, Zn and Sb presented a strong loading in PC2 (loading values > 0.75) (Zeng, Han & Yang, 2020). V and Sb presented a significant positive correlation in Fig. 4. Moreover, V, Zn and Sb presented the same spatial distribution pattern among rivers, such as the concentrations of V, Zn and Sb in HR affected by human activities were significantly higher than those in other rivers (Wu et al., 2019). A study in 2010 found that the concentrations of V and Sb were high in the upper HR, which was attributed to the input of human activities (Li & Zhang, 2010). Other studies showed that V, Zn and Sb were widely used in industry (Gao et al., 2020; Kravchenko et al., 2014). Therefore, though the three elements had their natural sources, we inferred that industrial emissions were the dominant factor for V, Zn and Sb in group 2.

Group 3 mainly contained As and Mo. These two metals were the main contributors to PC3 (Fig. 5) and presented a significant positive correlation (Fig. 4). The concentration of As was higher in QLR, DJR and XSR. QLR and XSR were located in the main urban area of Wuhan, and the high concentration of As might be related to urban activities. The DJR was located in the middle and lower reaches of the Yangtze River, with many plains and higher agricultural activities than other areas, so soil erosion caused by farming might be another important reason for the increase of As concentration in the water body (Wu et al., 2019). The concentration of Mo was also higher in QLR and XSR, so Mo might mainly be human input of urban activities. Previous studies showed that Mo was widely used in electronics, medicine and agriculture (Elbaz-Poulichet et al., 2006; Liang, 2020). Therefore, we attributed group 3 to the mixed input of urban and agricultural activities.

As described in the above analysis, Mn, Fe, Co and Ni were natural sources, while V, Zn, As and Sb were mainly anthropogenic sources. It was worth noting that Mo was present in group 1 and group 3 simultaneously, which indicated that the natural process and human activities influenced the change of Mo in water.

### Spatial distribution of heavy metal pollution index (HPI)

**Figure 6:**
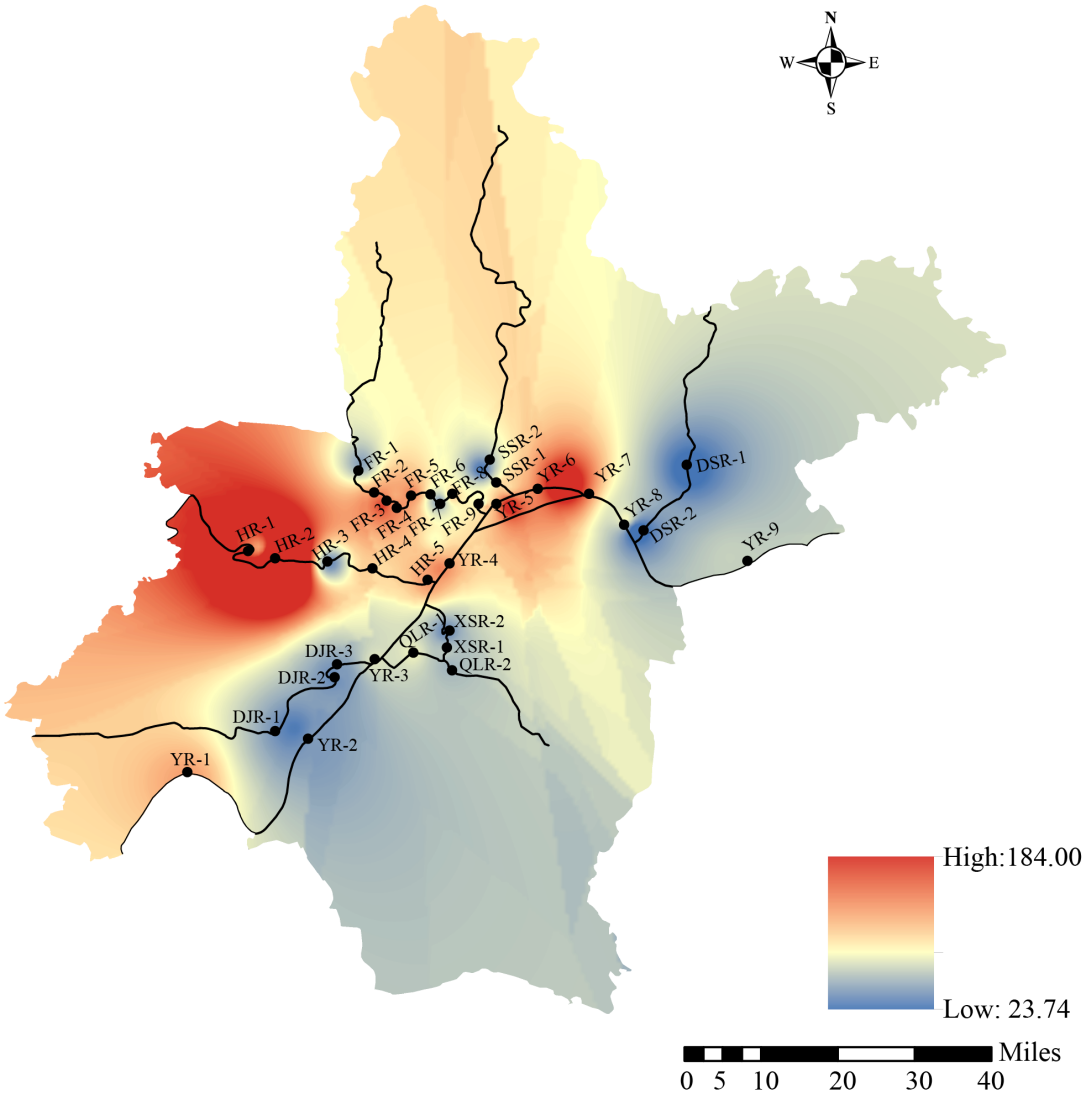
Spatial distribution of HPI.

In this study, HPI was used for describing the potential environmental risks of heavy metals in water. It calculated the spatial trend of the HPI based on nine heavy metals in the rivers (Fig. 6). HPI ranged from 23.74 to 184.0. The HPI values of all sampling points belonged to a high level (HPI > 30), except for the two sampling points of Daoshui River. The results showed that the ecological problems of heavy metals in river water were widespread (Tiwari et al., 2015). In addition, the contribution of Zn in the evaluation of HPI of rivers in Wuhan was not significant because of its lower weightage (Wi) value. Heavy metals such as Mn, Fe, Ni, As, Mo and Sb had higher weightage (Wi) values and higher HPI values, indicating that the higher concentration of these heavy metals in river water, the worse water quality (Bhaskar et al., 2020). It should be noted that As and Sb contribute most of the HPI values, which was consistent with their higher concentrations in the river. The spatial distribution of HPI values showed that the ecological risk of heavy metals around HR-2 was the highest, and the HPI value of this point was 184.0, which exceeded the value of 100, which was the critical pollution index value (Bhaskar et al., 2020). Overall, except for DSR-1 and DSR-2 sites, the ecological risk of heavy metals in all sampling sites was a severe problem. It might be due to industrial, agricultural and domestic activities (Wang et al., 2017).

### Human health risk assessment

The calculation results of HQ and HI values of heavy metals for adults and children are demonstrated in Table 4. The HQ_ingestion_, HQ_dermal_ and HI of all heavy metals evaluated were less than the threshold 1 we considered in this study for both adults and children. Results presented that the harm of nine heavy metals in rivers of Wuhan to residents was low. Moreover, we observed that children had higher HQ_ingestion_ and HQ_dermal_ than adults, indicating that heavy metals were more harmful to children. It was worth noting that the HI values of Sb, As and V were at least one order of magnitude higher than other heavy metals. Therefore, human health monitoring and environmental supervision agencies should pay special attention to the sources and exposure routes of Sb, As and V in rivers in Wuhan.

**Table 4:** Hazard quotient, dermal permeability coefficient and reference dose for heavy metals in rivers in Wuhan.

	HQ_ingestion_		HQ_dermal_		HI		K_p_^a^ cm/h	RfD_ingestion_^b^^c^ μg/kg/day	RfD_dermal_^b^^c^ μg/kg/day
	Adults	Children	Adults	Children	Adults	Children			
V	5.22E−02	7.80E−02	5.45E−02	1.61E−01	1.07E−01	2.39E−01	2 × 10^−3^	1	0.01
Mn	1.03E−03	1.54E−03	1.35E−04	3.98E−04	1.17E−03	1.94E−03	1 × 10^−3^	24	0.96
Fe	5.01E−04	7.48E−04	1.31E−05	3.86E−05	5.14E−04	7.87E−04	1 × 10^−3^	700	140
Co	5.66E−03	8.46E−03	5.91E−05	1.74E−04	5.72E−03	8.63E−03	4 × 10^−4^	0.3	0.06
Ni	1.59E−03	2.37E−03	4.15E−05	1.22E−04	1.63E−03	2.50E−03	2 × 10^−4^	20	0.8
Zn	1.92E−04	2.87E−04	3.01E−06	8.88E−06	1.95E−04	2.96E−04	6 × 10^−4^	300	60
As	3.40E−01	5.08E−01	4.33E−03	1.28E−02	3.44E−01	5.21E−01	1 × 10^−3^	0.3	0.123
Mo	1.25E−02	1.87E−02	3.44E−04	1.01E−03	1.28E−02	1.97E−02	2 × 10^−3^	5	1.9
Sb	2.93E−01	4.38E−01	7.66E−02	2.26E−01	3.70E−01	6.64E−01	1 × 10^−3^	0.4	0.008

**Notes:**

a United States Environmental Protection Agency (2004).

b Wang et al. (2017).

c Wu et al. (2019).

**Figure 7:**
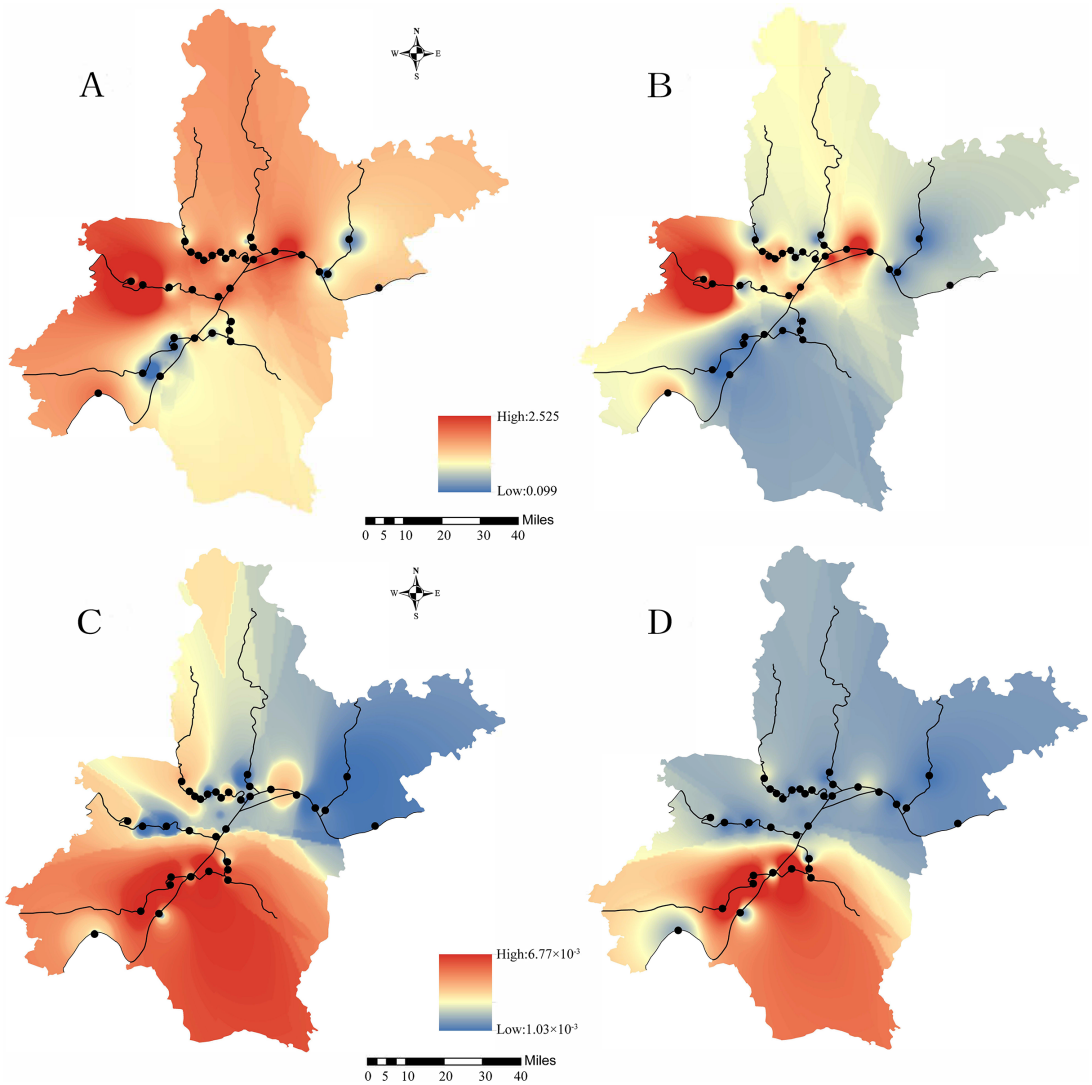
Spatial distribution of HI for Sb (A and B) and CR for As (C and D). (A), (C) Children; (B), (D) Adults.

The HI values of V and Sb and the CR values of As and their spatial distribution are shown in Table 5 and Fig. 7. For adults and children, the order of HI values of V in the rivers was HR>QLR>FR>XSR>YR>DJR>SSR>DSR, and the high-risk areas were HR, QLR, FR and XSR. The order of HI values of Sb was YR>HR>FR>SSR>XSR>QLR>DSR>DJR, and the high-risk areas were YR, HR, FR and SSR. In addition, the HI value in YR (0.843 for children), HR (0.808 for children) and FR (0.732 for children) were relatively close to 1. Therefore, Sb might have potential non-carcinogenic to human health, especially in the areas around the HR-2, YR-6, YR-7 and FR-3 (Figs. 7A and 7B). The HI value of As for children in QLR exceeded threshold 1, and that of adults was relatively close to 1. Moreover, the HI values of As in DJR (0.586 for adults, 0.886 for children) were relatively close to 1.Therefore, As might have potential non-carcinogenic risk to adults and children in these areas. The order of CR value of As in the rivers was QLR>DJR>XSR>HR>FH>YR>SSR>DSR. The CR values of As all exceeded the target risk (10^−4^) (Chen & Liao, 2006; Qu et al., 2018), which indicated that the carcinogenic risk of As to adults and children was widespread in the study area. Spatially, the areas around QLR-1 and DJR-1 have the highest risk (Figs. 7C and 7D). These findings deserve our grave concern about the carcinogenic effects of As in rivers in Wuhan.

**Table 5:** The HI values of V, Sb and As and CR values of As in rivers in Wuhan.

	V(HI)		Sb(HI)		As(HI)		As(CR)	
	Adults	Children	Adults	Children	Adults	Children	Adults	Children
Yangtze River	9.95E−02	2.23E−01	4.65E−01	8.34E−01	2.78E−01	4.21E−01	1.24E−03	1.85E−03
Dongjin River	7.21E−02	1.61E−01	1.31E−01	2.36E−01	5.86E−01	8.86E−01	2.60E−03	3.88E−03
Qingling River	1.11E−01	2.49E−01	2.07E−01	3.72E−01	7.21E−01	1.09E+00	3.20E−03	4.77E−03
Xunsi River	1.05E−01	2.35E−01	2.27E−01	4.07E−01	3.75E−01	5.67E−01	1.66E−03	2.48E−03
Han River	1.72E−01	3.85E−01	4.50E−01	8.08E−01	2.91E−01	4.40E−01	1.29E−03	1.93E−03
Fu River	1.10E−01	2.47E−01	4.08E−01	7.32E−01	2.90E−01	4.39E−01	1.29E−03	1.93E−03
Sheshui River	7.18E−02	1.61E−01	2.94E−01	5.28E−01	2.67E−01	4.04E−01	1.19E−03	1.77E−03
Daoshui River	6.00E−02	1.34E−01	1.36E−01	2.45E−01	2.59E−01	3.91E−01	1.15E−03	1.71E−03

According to the comprehensive results of the HI and CR index, the high-risk areas of rivers in Wuhan were summarized as follows: (1) As pollution was a significant threat to human health and the high-risk areas were QLR and DJR. (2) The high-risk areas of V were HR, QLR, FR and XSR, especially in HR, QLR-1 and XSR-1. (3) The high-risk areas of Sb were YR, HR and FR, especially in YR-6, YR-7 and HR-2.

According to the risk assessment results, As and Sb were the primary pollutants in rivers in Wuhan, which were consistent with the comparison results of drinking water standards (Table 2). The side effects of heavy metals have been widely reported, such as V has a toxic impact on the human digestive system, blood, nervous system and immune system (Chlubek et al., 2012; Rawal et al., 1997). Sb may cause discomforts such as nausea, vomiting, anorexia, abdominal pain and gastric ulcer (Saerens et al., 2019; Sundar & Chakravarty, 2010). Moreover, excessive As intake may cause cancers (liver cancer, lung cancer, bladder cancer) and other side effects (hypertension, neuropathy, diabetes, tibial disease) (Wu et al., 2009b; Xu & Wang, 2020). Therefore, we should pay special attention to the potential non-carcinogenic risk of V and Sb and the potential carcinogenic risk of As. It is necessary to take relevant measures to prevent excessive heavy metals from entering rivers, and maintain a healthy water ecosystem and provide water resources guarantee for the economic development of Wuhan.

However, there was uncertainty in the description of health risks for heavy metals, which had been pointed out by US EPA and other relevant studies (Qu et al., 2018; United States Environmental Protection Agency, 2009; Wu et al., 2009b). Water and skin contact factor (K_p_), different exposure conditions caused by different ages and receptors, temporal changes of pollutant concentration and daily water intake cannot be quantified, which led to methodological uncertainty. Furthermore, the US EPA is the primary source of parameters in this study, which might not apply to the Chinese. Consequently, it is necessary to clarify the risk characteristics further and improve the risk assessment method by investigating river risk levels in Wuhan.

## Conclusions

The spatial distribution of dissolved heavy metals in Wuhan had great spatial heterogeneity. Different distribution characteristics of dissolved heavy metals caused by human activities (industrial, agricultural, domestic) in different regions of Wuhan. The results of CA and PCA showed that Mn, Fe, Co, Ni and Mo were attributed to the geological sources of the basin, and V, Zn and Sb were controlled by industrial activities, while As was mainly dominated by the mixed input of urban and agricultural activities.

In general, the concentrations of all heavy metals in Wuhan river water were within the standard range of China’s drinking water. However, Sb in YR and HR exceeded the drinking water guidelines set by WHO, US EPA and China EPA, and As in DJR and QLR were close to the three drinking water guidelines, which required special attention. The heavy metal pollution index results showed that As and Sb contributed most of the HPI values, which was consistent with high concentrations in the rivers, indicating that these two heavy metals’ ecological risk was high. Non-carcinogenic risk and carcinogenic risk of heavy metals were analyzed by HI and CR. The results showed that V and Sb had potential non-carcinogenic risk and As might have a potential carcinogenic risk to adults and children in the study area, while As might have a potential non-carcinogenic risk for children around QLR. The results showed that As and Sb were the primary pollutants in rivers in Wuhan. We should prevent them from damaging the aquatic ecosystem and provide water resources guarantee for the economic development of Wuhan. It is necessary to develop a long-term monitoring scheme for As and Sb, which can effectively control and manage the As and Sb pollution in rivers in the corresponding areas.

## Supplemental Information

10.7717/peerj.11853/supp-1 Supplemental Information 1 Comparison of heavy metal concentrations (μg/L) in rivers in Wuhan with other rivers in the world.

10.7717/peerj.11853/supp-2 Supplemental Information 2 Data of measured dissolved heavy metal concentrations, physicochemical characteristics, and date of sampling in Yangtze River, Dongjing River, Han River, Qingling River, Xunsi River, Fu River, Sheshui River and Daoshui River for Figs. 2 and 3.

## Additional Information and Declarations

### Data Availability

The following information was supplied regarding data availability: Data of measured dissolved heavy metal concentrations, physicochemical characteristics, and date of sampling are available in the Supplemental Files.
