# Supplementary material for: Distribution, source, water quality and health risk assessment of dissolved heavy metals in major rivers in Wuhan, China
Source: PeerJ. 2021 Jul 27;9:e11853. doi: 10.7717/peerj.11853 (PMC8323599; doi:10.7717/peerj.11853)
Supplement: Supplemental Information 1 [file peerj-09-11853-s001.docx]

Table S1 Comparison of heavy metal concentrations (μg/L) in rivers in Wuhan with other rivers in the world.

| Rivers | **V** | **Mn** | **Fe** | **Co** | **Ni** | **Zn** | **As** | **Mo** | **Sb** | reference |
| --- | --- | --- | --- | --- | --- | --- | --- | --- | --- | --- |
| This study | 1.91 | 0.90 | 12.80 | 0.06 | 1.16 | 2.10 | 3.72 | 2.28 | 4.29 |  |
| Huai River, China |  | 49.02 | 440.65 | 42.49 | 46.19 | 10504.19 |  |  |  | Wang et al., 2017 |
| Xiangjiang River, China |  |  |  |  |  | 84.57 | 12.24 |  |  | Zeng et al., 2015 |
| Dan River, China | 4.87 | 6.72 | 2.71 | 0.15 | 1.68 | 7.83 | 7.26 |  | 27.82 | Meng et al., 2016 |
| Tarim River, China | 1.06 | 16.51 | 61.85 | 0.10 | 1.79 | 7.11 | 3.07 |  | 0.45 | Xiao et al., 2014 |
| Zhujiang River，China | 2.66 | 5.84 |  | 0.12 | 4.43 |  |  | 1.89 |  | Zeng et al., 2018 |
| To Lich River, Vietnam |  | 216.20 |  |  | 7.60 | 51.10 | 39.10 |  |  | Thuong et al., 2013 |
| Haraz River, Iran |  | 116.00 |  |  | 22.40 | 52.75 | 55.35 |  |  | Nasrabadi, 2015 |
| Benue River, Nigeria |  | 181.00 | 751.00 |  |  | 78.70 |  |  |  | Eneji et al., 2012 |
| Subarnarekha River, India | 14.10 | 12.00 | 133.80 | 0.57 | 25.20 |  | 5.41 |  |  | Singh and Giri, 2014 |
| Tigris River, Turkey |  | 467.00 | 388.00 | 111.00 | 72.00 | 37.00 | 2.35 |  |  | Varol and Şen, 2012 |
| Seine River, France |  | 3.92 |  |  |  |  | 0.64 | 0.68 |  | Elbaz-Poulichet et al., 2006 |
| Hawkesbury-Nepean River, Australia |  | 52.00 | 268.00 | 0.24 | 0.26 | 0.88 |  |  |  | Markich and Brown, 1998 |
| Indin River, Canada | 0.01 | 1.89 | 50.00 | 0.02 | 0.64 | 1.52 | 0.35 |  | 0.01 | Gaillardet et al., 2003 |
| Amazon, USA | 0.70 | 50.73 |  | 0.18 | 0.74 | 0.45 |  | 0.18 | 0.06 | Gaillardet et al., 2003 |
| Trinity River, USA | 2.05 | 4.15 | 5.81 | 0.16 | 2.07 |  |  |  |  | Warnken and Santschi, 2009 |
| World average | 0.71 | 34.00 | 66.00 | 0.15 | 0.80 | 0.60 | 0.62 | 0.42 | 0.07 | Gaillardet et al., 2003 |
